# Economic evaluations of interventions for the treatment and management of non-communicable diseases in humanitarian contexts: A scoping review

**DOI:** 10.1371/journal.pgph.0006740

**Published:** 2026-11-11

**Authors:** Asmaa Elnajar, Matthew Allsop, Maryam Ba-Break, Nour Kasasbeh, Bryony Dawkins

**Affiliations:** 1 Academic Unit of Health Economics, Leeds Institute of Health Sciences, University of Leeds, Leeds, United Kingdom; 2 Academic Unit of Palliative Care, Leeds Institute of Health Sciences, University of Leeds, Leeds, United Kingdom; 3 Nuffield Centre for International Health and Development, Leeds Institute of Health Sciences, University of Leeds, Leeds, United Kingdom; Walter Sisulu University, SOUTH AFRICA

## Abstract

Humanitarian crises profoundly disrupt health systems and limit access to healthcare. People with non-communicable diseases (NCDs) are among the most vulnerable groups affected. As humanitarian interventions often focus on acute health needs, NCDs are often overlooked in financing and service delivery. In such contexts, efficient strategies are crucial for maximising health benefits and ensuring equitable service delivery. Health economic evaluations provide decision-makers with evidence on optimal resource allocation. Given the high burden of NCDs and the uncertainty about the effectiveness of their management in such contexts, this review aims to map available evidence from studies that utilised health economic approaches to assess NCD programmes implemented in humanitarian contexts. Systematic searches were conducted across seven electronic databases: Ovid Medline, Global Health, Embase, Scopus, Web of Science, CAB, and EconLit, alongside grey literature. Eligible studies were full economic evaluations (cost-effectiveness, cost-utility, and cost-benefit analyses) of NCD interventions in humanitarian contexts. Of the 6911 deduplicated records screened, seven studies met the inclusion criteria; a structured Excel sheet charted key study characteristics. The included evidence comprised four studies from conflict-affected lower-middle-income countries, and three from natural disaster settings in high-income countries. No studies from low-income countries met the inclusion criteria. NCDs addressed included mental health (n = 4), asthma (n = 2), and congestive heart failure (n = 1). One study addressed broader NCD-related public health interventions, including cancer screening and management, and incorporated diabetes into an NCD burden model without cost-effectiveness analysis. Methodological approaches varied, including simulation/microsimulation (n = 3), trial-based economic evaluations (n = 3), and a Markov model (n = 1). The review highlights limited and fragmented evidence to support decision-making and long-term planning, particularly in low-income settings. Further research is needed to understand how health economic evidence for NCDs can be generated, interpreted and used within humanitarian decision-making systems, and to support allocation of scarce resources for people living with NCDs during crises.

## 1. Introduction

Despite the high morbidity and mortality associated with non-communicable diseases (NCDs), especially during crises, NCDs often receive low priority compared with other humanitarian interventions and acute cases [1,2]. People with NCDs require long-term, sustained health services to prevent further complications and premature deaths. During emergencies, a disrupted health system and limited resources increase this group’s vulnerability, heightening the risk of complications, disability, and premature death [3,4]. As a result, efficient strategies are necessary to inform decision-makers on how to allocate scarce resources optimally [5]. Health economic evidence can provide decision-makers with efficient tools to ensure the accessibility and sustainability of health services [6,7]. However, it remains unclear how much such evidence is available and used by decision-makers in humanitarian contexts.

NCDs are long-lasting conditions with lasting consequences, including cancers, cardiovascular diseases, diabetes, and chronic respiratory diseases [8]. Globally, one in five people suffers from at least one NCD [2]. In 2021, NCDs accounted for 75% of all deaths, of which 82% were premature deaths recorded in low- and middle-income countries [2,8]. NCD management is a big challenge not only for people with NCDs but also for healthcare providers and caregivers, especially in resource-limited settings, due to financial and human resource constraints, inadequate health supplies, a disrupted supply chain, including essential medications, lack of care continuity, poor information systems, and a lack of political commitment and coordinated action [1,2].

In humanitarian contexts, NCDs compete with other health threats, such as communicable diseases, as well as with other humanitarian needs, such as food and shelter [9]. However, the focus during crises is primarily on acute needs, such as maternal and newborn health, conflict-related injuries, and infectious disease outbreaks [2], often leaving NCD programmes overlooked and relevant global humanitarian programmes underfunded [10].

Humanitarian contexts arise from natural (e.g., earthquakes, wildfires, pandemics) or man-made (e.g., armed conflict, industrial crises) events and threaten the health, safety, and well-being of large groups of people [11,12]. Generally, humanitarian crises worldwide are becoming more complex, frequent, multifaceted, and protracted [4]. In 2025, over 300 million people are estimated to be affected by a humanitarian crisis [13]; of these, 56,000 civilians were killed in conflict-related events in the first 10 months of 2025 [14]. Currently, one in every eight people lives in a fragile and conflict-affected context, and this number is expected to rise as a result of extreme weather conditions and intensifying armed conflict [2].

Over the past decade, the scale and complexity of health interventions in humanitarian contexts have increased exponentially, while donor budgets have become increasingly restrictive. Consequently, optimal fund allocation, ensuring that resources are directed to the right places, in the right way, and at the right time, has become fundamental [15,16]. Economic evaluation methods such as cost-effectiveness, cost-utility, and cost-benefit analyses provide tools, theories, and structured approaches to determine value for money, thereby supporting better resource allocation [17]. In humanitarian crises, economic evaluation is vital not only for health policymakers but also for donors, as it enables the identification of the most effective interventions amid scarce resources. It can assist real-time decision-making, support the prioritisation of constrained resources under uncertainty and time pressure, provide evidence on the economic and financial implications of various interventions [18], support long-term budget planning, and facilitate the creation of new funding channels [19].

Available evidence from economic evaluations of NCD service delivery in emergency and humanitarian contexts appears to have received limited systematic synthesis regarding the extent of the evidence base, methodological approaches and the coverage of different NCDs [20]. Identified reviews have generally focused on broader humanitarian health programmes or specific geographical regions. For example, Makhani et al. [18] highlighted the limited evidence of full economic evaluation in health-related humanitarian programmes in low- and middle-income countries. However, Makhani et al. study was confined to general health programmes and restricted geographically to low- and middle-income countries. As such, there is a need for a focused synthesis of economic evidence covering all NCDs across humanitarian contexts globally.

### 1.1. The scoping review objective and question(s)

Given several limitations of synthesised evidence in this field, this scoping review sought to systematically map the existing literature linking humanitarian contexts, NCDs and economic evaluation. This was achieved by addressing several interrelated questions. Firstly, it aims to identify the types of humanitarian contexts across different geographical regions and country income levels that have published health economic evaluations of NCD management. In addition, it aims to examine the thematic focus and methodological characteristics of published economic evaluations, including the types of economic evaluation approaches and analytical perspectives applied to evaluate NCD programmes and interventions in humanitarian contexts across low-, middle-, and high-income countries. It also aims to explore the most commonly evaluated NCDs in such contexts, identify evidence gaps and limitations in applying health economic evaluation to NCD interventions in humanitarian contexts, and explore contextual variations and commonalities across humanitarian contexts.

The conceptual framework underpinning this review integrates three interrelated concepts: humanitarian contexts, NCD interventions and health economic evaluation. Humanitarian crises can collapse health systems, restrict access to medications and essential NCD services such as screening and diagnosis and increase pressure on already limited resources. These challenges often create difficult priority-setting decisions regarding the delivery and financing of NCD interventions. In such contexts, health economic evaluation provides a structured approach to assessing the costs and consequences of alternative interventions, generating robust evidence to support resource allocation and policy decisions. Fig 1. illustrates the relationship among these concepts, showing how humanitarian crises disrupt NCD management and how economic evaluation can support decision-making and resource allocation to improve NCD management.

**Fig 1:**
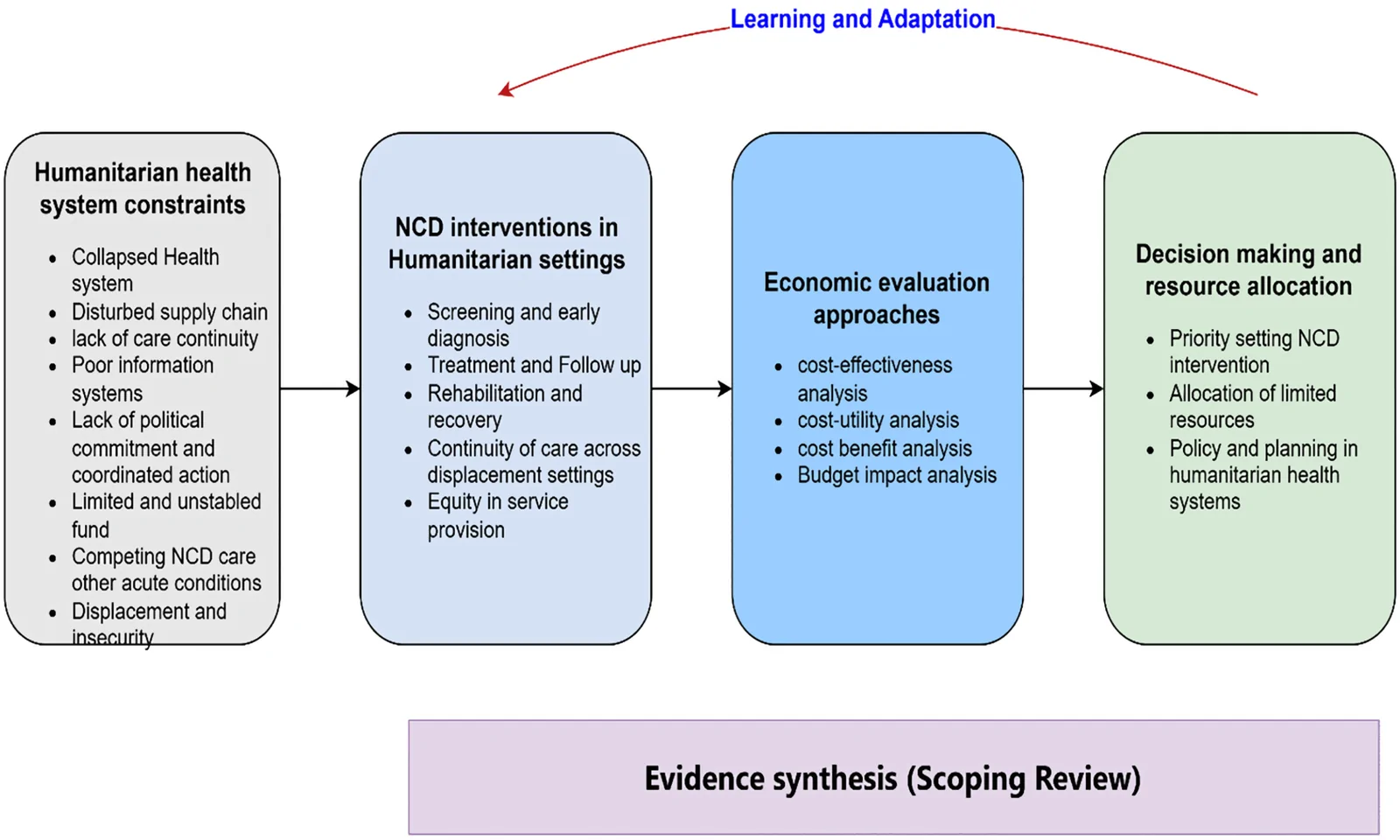
Conceptual framework for economic evaluation of NCD interventions in humanitarian contexts.

## 2. Materials and methods

The scoping review protocol was pre-registered with the Open Science Framework (OSF) (DOI: https://doi.org/10.17605/OSF.IO/PR6TF) [21].

The scoping review adhered to the Joanna Briggs Institute methodological guidance for scoping reviews, and reporting was guided by the Preferred Reporting Items for Systematic Reviews and Meta-Analyses - Scoping Reviews extension (PRISMA-ScR) [22,23]. The PRISMA-ScR checklist is provided in the Supplementary Material, S1 Checklist.

The review’s focus is outlined using the SPICE framework (Setting, Perspective, Intervention, Comparator, and Evaluation), which informed the development of the review question, search strategy and eligibility criteria. It is well suited to qualitative and mixed-methods evidence searches, as well as to evaluating the outcomes of projects or interventions. Although the Joanna Briggs Institute (JBI) methodology recommends the Population, Concept, Context (PCC) framework for scoping reviews, SPICE is considered more appropriate for this review and it maps well onto PCC, ensuring consistency with JBI methodology.

Health economic evaluations are inherently decision-oriented and assess different interventions across settings from specific perspectives, in relation to defined outcomes. These dimensions are explicitly represented in the SPICE framework [24]. Although PCC provides broader guidance for concept mapping, it does not capture common health economics features such as intervention comparisons, analytical perspectives, and evaluation outcomes. The SPICE framework was operationalised as follows: Settings referred to humanitarian contexts; Perspectives referred to populations, decision-makers, and health system actors whose viewpoints shape decision-making and resource allocation; Intervention referred to NCD-related interventions; Comparator, where applicable, referred to alternative interventions, standard care, or no intervention; and Evaluation referred to outcomes and measures reported in health economic evaluations.

### 2.1. Eligibility criteria

For study selection, a humanitarian context was defined as a setting affected by natural disasters (earthquakes, floods, wildfires) or conflict-related crises, such as armed conflict, in which the health, safety, or well-being of a large group of people is acutely threatened [11,12]. Both acute and protracted crises were eligible. In line with the OCHA and SPHERE frameworks, which distinguish between complex emergencies and public health emergencies and focus only on natural disasters and conflict settings [25,26], pandemics and disasters from other causes (e.g., technological or nuclear disasters) were excluded. This decision is also supported by conceptual and methodological reasons for excluding pandemics and technological disasters. Conceptually, pandemics and technological disasters represent distinct humanitarian emergencies with different response frameworks, government structures, and healthcare priorities compared with conflict-related crises and natural disasters. They may create humanitarian needs and response mechanisms that may differ substantially from those associated with acute care disruption, infrastructure damage, displacement, and insecurity, commonly observed in conflict and natural disaster settings [26,27]. Methodologically, excluding these contexts reduces heterogeneity in intervention types, evaluation methods, and outcome measures, improving consistency and enabling meaningful synthesis of the evidence. In terms of types of evaluation, partial economic evaluations, such as cost analyses, were ineligible for inclusion in this review. Only full economic evaluations that considered both costs and consequences to compare different NCD programmes or interventions were included. This will enable a full understanding of the quality and quantity of health economic evaluations assessing NCD interventions conducted in humanitarian contexts [18].

Studies addressing interventions for the management of NCDs in humanitarian contexts, including screening, diagnosis, treatment, or rehabilitation, were included. Studies addressing a broad range of predefined NCDs were eligible for inclusion, including but not limited to cardiovascular diseases, cancers, diabetes, stroke, chronic respiratory diseases, mental health and chronic neurological disorders, and chronic kidney diseases [2,28]. No restrictions were applied to language or year of publication during the search and screening process to ensure inclusion of all relevant, available reports. In order to include articles in any language, the intention was to use translation services (e.g., Google Translate), but in practice this was not required as all records identified had their title and abstract available in English, even when the full article was published in another language. As such, non-English records were screened based on the English titles and abstracts provided by the indexed databases. Following title and abstract screening, no non-English records met the inclusion criteria for full-text review, and so further translation software and additional language expertise were not required.

Table 1 lists the eligibility criteria used to guide the review process.

**Table 1:** Inclusion and exclusion criteria.

Category	Inclusion criteria	Exclusion criteria
**Study setting**	Studies conducted in humanitarian contexts affected by natural disasters (earthquakes, floods, wildfires) and/or armed conflicts. Settings with either long-term crises or acute disasters could be included.	Studies conducted in contexts of pandemics, technological disasters (e.g., nuclear and industrial disasters) and radiation emergencies, provided there is no compounding natural disaster or armed conflict.
**Population**	Populations living in humanitarian contexts (as defined above) who are living with or at risk of key non-communicable diseases (as defined below).	Populations originating from humanitarian contexts but who have migrated out and are no longer residing within an eligible active humanitarian setting Populations presenting exclusively with health conditions outside the review’s predefined NCD scope (e.g., acute trauma/injuries), or maternal/child health conditions without NCD comorbidity.
**Type of study**	Full economic evaluation studies comparing two or more programme alternatives, examining both costs and consequences. Studies in which an economic evaluation (as per the definition above) is conducted and reported as part of a larger study.	Partial economic evaluations (e.g., cost-only analyses, cost-description studies), and clinical trials evaluating clinical effectiveness alone, without integrated health economic components. Reviews, protocols, or studies lacking sufficient quantitative data to extract cost/consequence values
**Types of intervention**	Eligible NCD conditions include, but are not limited to, cardiovascular diseases, cancers, chronic respiratory diseases, diabetes, mental health conditions, chronic kidney diseases and chronic neurological disorders [2,28]. Interventions for the screening, diagnosis, treatment, or management of NCDs.	Broad, non-targeted public health interventions or general humanitarian aid packages in which specific, distinct NCD management components cannot be isolated and evaluated independently.
**Outcome of intervention**	Studies that include a relevant health outcome. This can be a directly observed health outcome (e.g., HbA1c reduction or lives saved), or a composite measure of quality and length of life (e.g., quality-adjusted life years (QALYs) or disability-adjusted life years (DALYs)).	Studies reporting solely on financial expenditures, operational resource utilisation, or process metrics (e.g., number of clinic visits) without measuring clinical endpoints, quality of life (QALYs/DALYs), or mortality.

### 2.2. Information sources and search strategy

A comprehensive literature search was conducted using seven electronic bibliographic databases, relevant to the subject area: MEDLINE (via Ovid), Embase (via Ovid), Global Health (via Ovid), Web of Science, CAB (via Ovid), Scopus, and EconLit. Grey literature was sought from the CAB database and by searching relevant organisation websites, including the World Health Organisation and ReliefWeb. ReliefWeb covers key sources from different UN agencies and from international and local NGOs [29]. The search of the seven bibliographic databases was structured around three main concepts: humanitarian context, noncommunicable diseases, and health economic evaluation. Grey literature searching was conducted using one or two of the three main concepts as search terms, while the remaining concepts were applied as filters via the available options in the website’s drop-down menu. In addition, hand-searching of the reference lists of included studies was undertaken.

### 2.3. Search

The full search strategy for these concepts is provided in the supplementary material text of the S1 Text. The search strategy was developed and reviewed by an expert Information Specialist. The search was adapted for each database, considering the database-specific MeSH terms and Boolean operators. Initial searches were undertaken in July 2025, with an updated search conducted in July 2026 ahead of publication, to include recent publications since the initial searches and to add evidence from the EconLit database, which was not searched in the first round.

The search strategy used to search grey literature is available in the supplementary material table in S1 Table.

### 2.4. Selection of sources of evidence

All search results from the seven databases were imported into EndNote, version 21 (Clarivate, Analytics, Philadelphia, PA, USA) for reference management and deduplication [30]. Following deduplication, screening was conducted using Rayyan, a web-based systematic review screening platform [31]. Pilot title and abstract screening was conducted on fifty randomly selected records. During piloting, no necessary refinements to the inclusion and exclusion criteria were required. Next, two independent reviewers (AE, NK) conducted full title and abstract screening. Article selection followed the clearly defined eligibility criteria outlined above. Reviewers discussed and resolved any disagreements during this phase of screening until consensus was reached. All records that did not meet these criteria were excluded.

The next stage was full-text screening for eligibility. The full text of the report identified as potentially eligible was screened independently by two reviewers (AE, NK). During this phase, a third reviewer was consulted to assist in making the final decision after full-text screening. Records deemed not relevant or ineligible after the double review and wider team consensus discussions were excluded, with clearly documented justifications.

In the event that multiple reports were identified describing the same study, each was included, although the extracted data were linked to a single study only to avoid double-counting during synthesis.

### 2.5. Data charting

A predefined data extraction framework was developed deductively, informed by the review’s main objectives, key elements of health economic evaluations, scoping review methodology guidance, and the review of relevant literature. The framework’s main domains include study identification, study characteristics, intervention and comparators, outcome measures, economic evaluation characteristics, key findings, and quality assessment-related information. The draft framework was reviewed and refined by the co-authors until the final extraction categories were reached. The data charting and extraction framework is available in the supplementary material as S2 Table. Data were charted primarily by the corresponding author (AE) from the included studies, entered into a Microsoft Excel sheet, and then reviewed by the second reviewer (NK). This Followed the Joanna Briggs Institute methodology for scoping reviews [32], a structured data charting form was developed in Microsoft Excel to capture relevant information from each study. Extracted data included study characteristics, such as author name, year of publication, journal name, DOI, setting name and type, study design, sample size, population characteristics, study period, and intervention and comparator details where applicable. Information related to economic evaluation was also collected, including the type of economic evaluation (e.g., cost-effectiveness analysis, cost-utility analysis), cost components included, time horizon, perspectives, discount rate, currency and price year, whether sensitivity analysis and quality assessment were performed, and funding sources and conflicts of interest. In addition, data were extracted on the measured outcome and key findings, including primary outcomes (e.g., cost-effectiveness, QALYs, DALYs), secondary outcomes (e.g., health outcomes, quality of life, resource use), authors’ conclusions, and limitations noted by the authors.

### 2.6. Critical appraisal of included studies

Although critical appraisal is not a mandatory component of scoping reviews, the methodological quality of the included economic evaluations was assessed using the Drummond 10-item checklist [17]. This checklist was designed to guide the reporting of economic evaluations and provides guidance on what should be reported in relation to each aspect of the economic evaluation. It is used as a tool to critique the methods and reporting of each relevant aspect of the evaluation: 1) the research question; 2) the description of the study/intervention; 3) the study design; 4) the identification, 5) measurement, and 6) valuation of costs and consequences; 7) whether discounting was carried out; 8) incremental analysis; 9) presentation of results, including uncertainty and sensitivity analyses; and 10) discussion of results in the context of policy relevance and existing literature [17]. Because this scoping review maps existing evidence, this appraisal assessed the quality of the economic evidence rather than excluding studies.

### 2.7. Data analysis

Data analysis was conducted manually in Microsoft Excel, in line with the data extraction/analysis framework described above and available in the supplementary material table (S2 Table). Data extracted from the included studies were summarised for each study using descriptive statistics and narrative synthesis. Descriptive statistics, including counts and frequencies, were used to summarise study characteristics, such as geographical distribution, humanitarian context type, intervention type and economic evaluation approaches. Narrative synthesis was undertaken to synthesise the data, identify key themes and patterns, and summarise the findings from the included records. Any decisions regarding data categories and synthesis were discussed among the review’s authors and resolved by mutual consensus.

## 3. Results

A total of 11,060 records were identified and extracted from the seven databases. The study selection process is depicted in the PRISMA flow diagram [33] (Fig 2).

**Fig 2:**
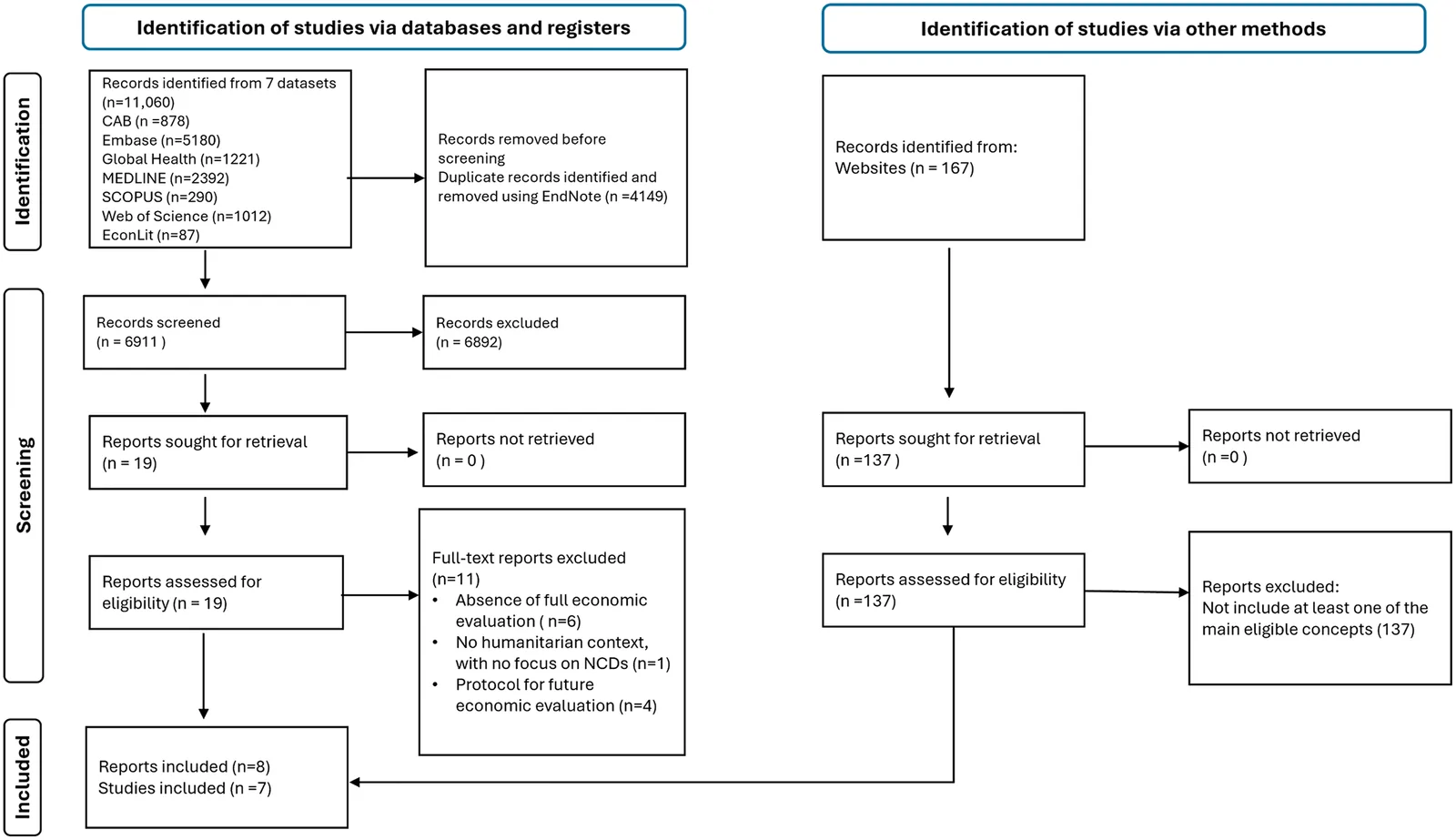
Study selection process. PRISMA 2020 flow diagram for new systematic reviews.

Following deduplication, 6911 records underwent title and abstract screening, of which 6892 were excluded. Exclusions at this stage were primarily due to the following reasons (exclusion reasons were not mutually exclusive): records conducted in non-humanitarian contexts (n = 1009), records focusing on a humanitarian context related to a pandemic (n = 352), and other ineligible crises, e.g., technological disasters (n = 1). A substantial number of records were excluded because they didn’t include a health economic evaluation or reported only a partial economic evaluation (n = 1959). Additional exclusions included records evaluating interventions addressing health conditions other than NCDs (n = 741) and records that didn’t meet the inclusion criteria (n = 1577). Other reasons for exclusion included clinical trials focused solely on the clinical effectiveness of medical interventions (n = 411) and records examining the health of migrants residing in stable, non-humanitarian contexts (n = 789). We excluded approximately 1,480 records because their primary focus fell outside the review scope. These records addressed environmental health, housing, air pollution, and other NCD-related issues in humanitarian contexts and didn’t meet any inclusion criteria.

During title and abstract screening, a small number of non-English records (n = 14) were identified, including Persian, German, Spanish, Turkish, French, Chinese, Russian, Czech, and Japanese. However, English titles and abstracts were available for each of these through the databases, allowing screening without further translation. None met the eligibility criteria; therefore, no non-English-language records progressed to full-text screening.

Two reviewers independently screened the titles and abstracts of identified records to determine eligibility. For n = 7 records, there was an initial disagreement between the reviewers about eligibility. However, after discussing these records, the reviewers reached consensus to include them.

Nineteen records progressed to full-text assessment, undertaken by two independent reviewers. During the full-text screening, eight reports, representing seven studies, met the inclusion criteria, while eleven were excluded. No disagreements between the two reviewers were reported at this stage.

Full-text exclusions were primarily due to the absence of a full economic evaluation (n = 6), the lack of a clear definition of the humanitarian context or a focus on NCDs (n = 1), and being protocols for future economic evaluations (n = 4) (see the PRISMA diagram in Fig 2). One report identified through the search was an updated conference abstract [34] on an already included study [35]. The abstract provided additional information and an extended study period. As this was a report on the same study, it was linked to the original publication to prevent duplication and incorporate the additional information [36]

Although hand-searching the reference lists of included studies identified some potentially eligible records, they were all duplicates of records already identified in the database search, so this process did not yield any additional eligible studies.

The grey literature search identified 167 records, including 123 from ReliefWeb and 44 from WHO IRIS. On ReliefWeb, searches used NCD-specific terms or specific NCD conditions, where applicable, and applied the “Humanitarian Financing” theme filter, the most relevant available category. On WHO IRIS, searches combined relevant health economics terms, including economic evaluation and cost-effectiveness, with subject headings for NCDs or chronic diseases for emergencies or disaster planning, as shown in S1 Table. After removing 30 duplicate records, 137 were screened. However, no grey literature reports met the full inclusion criteria; therefore, all were excluded because they did not report eligible economic evaluations of NCD interventions in humanitarian contexts.

### 3.1. Study characteristics

Table 2 Summarises the main characteristics of the seven included studies. The full data extraction table is available in the supplementary material as S2 Table, and the results of the quality appraisal are available in the S3 Table.

**Table 2:** Study characteristics of included studies.

Author	Study title	Country Income level	NCD category	Health economic evaluation approach	Modelling approach	Intervention	Health economics measure
Natural crisis-affected settings _ High-income countries							
Gregory H. Cohen et al. (2017) [37]	Comparison of Simulated Treatment and Cost-effectiveness of a Stepped Care Case-Finding Intervention vs Usual Care for Posttraumatic Stress Disorder After a Natural Disaster	High- income - (USA-New York City)	Mental disorders	Cost-effectiveness analysis cost per Disability Adjusted Life Years avoided and Post-traumatic stress disorder-free days)	Simulation- based modelling	A comparison of stepped care (SC) vs Usual care (UC) on treatment effectiveness and incremental cost-effectiveness in the 2 years after a natural disaster	10-year incremental cost-effectiveness. The 2-year reduction in the prevalence of PTSD among the full population
Amin Adibi et al. (2024) [35]	High Efficiency Particulate Air Filters for Preventing Wildfire-Related Asthma Complications: A Cost-Effectiveness Study	High- income - (Canada-British Columbia province)	Asthma	Cost-effectiveness analysis (cost per Quality Adjusted Life Years gained)	Markov model	High-Efficiency Particulate Air (HEPA) Filters with government rebates compared with Usual care without subsidised use.	HEPA filter rebates can be a cost-effective intervention to reduce acute asthma complications in wildfire-prone areas of the province.
Rappold et al. (2014) [38]	Forecast-Based Interventions Can Reduce the Health and Economic Burden of Wildfires	High- income - (USA, North Carolina)	Asthma, Congestive Heart Failure	Cost-benefit analysis (reduction in emergency department visits and premature mortality)	Simulation- based model	Forecast-based public health advisories triggered by PM2.5 thresholds (20, 35, 50 μg/m³) at varying compliance levels compared with no intervention	Health and economic outcomes Forecast-based interventions are effective and economically beneficial, especially when triggered early and followed with high compliance
Conflict-affected settings- lower- and middle-income countries							
Racha Abi Hana et al. (2017) [39]	Cost-Effectiveness of Digital Mental Health Versus Usual Care During Humanitarian Crises in Lebanon: Pragmatic Randomised Trial	Lower- Middle- income (Lebanon)	Mental disorders	Cost-effectiveness analysis (along with Randomised Controlled Trials)	Randomised Controlled Trial	Step-by-Step (SbS) – guided digital mental health intervention compared to Enhanced Usual Care (EUC)	Step by Step had an 80% probability of being cost-effective at a willingness-to-pay of $220 per additional treatment response and $840 per additional remission. SbS does not cost more than EUC, but it is associated with cost savings as SbS participants become more productive, thereby offsetting their healthcare costs.
Sanjay Basu et al. (2024) [40]	Reducing non-communicable diseases among Palestinian populations in Gaza: A participatory comparative and cost-effectiveness modelling assessment	Lower-middle income (Gaza)	Multiple public health interventions	Cost- effectiveness analysis (cost per Disability Averted Life Years)	Microsimulation model	Multiple public health and healthcare interventions (e.g., tobacco bans, asthma treatment, and cancer screening and treatment) compared with no intervention	The interventions with an incremental cost-effectiveness ratio less than three times the GDP per capita of Palestine per DALY averted for the included intervention
Wei-Lun Chang et al. (2018) [41]	Economic evaluation of bio-psycho-social intervention for comorbid disorders in a traumatised population in post-war Kosovo	Lower- middle- income (Kosovo)	Mental disorders	Multiple approaches: cost-effectiveness analysis, cost-utility analysis, and partial cost-benefit analysis	Randomised Controlled Trials	A 10-week multidisciplinary rehabilitation programme, comprising physiotherapy, biofeedback-supported psychotherapy and social support, compared with no intervention	The intervention showed socio-economic benefits but was above the WHO cost-effectiveness threshold for QALYs in the short term. i.e. the programme is not cost-effective in the short term per the WHO threshold, but potentially cost-effective over 4–5 years
Syed Usman Hamdani et al. (2020) [42]	Cost-effectiveness of WHO Problem Management Plus for adults with mood and anxiety disorders in a post-conflict area of Pakistan: randomised controlled trial	Lower-middle income (Pakistan)	Mental disorders	Cost-effectiveness analysis (Cost per unit improvement in anxiety/depression score/ Cost per successfully treated depression case)	Randomised Controlled Trials	Problem Management Plus (PM+) intervention compared with enhanced usual care.	The PM+ intervention was more effective but also more costly than EUC in reducing symptoms of anxiety and depression, and improving functioning in adults impaired by psychological distress in a post-conflict setting in Pakistan.

The seven included studies were published between 2014 and 2024. Four studies were conducted in conflict-affected settings [39–42]. Cost-effectiveness analysis was the most commonly used approach, adopting both healthcare and societal perspectives. Mental health conditions were the most commonly evaluated, followed by asthma.

Funding for the eligible studies was reported in six of them and was provided primarily by governmental research agencies, international development funders, philanthropic foundations, and public-sector organisations.

### 3.2. Quality appraisal

Assessment using the Drummond 10-item checklist, which evaluates the methodological quality and reporting of economic evaluations across ten key domains, indicated that the included studies met most appraisal criteria, as shown in S3 Table: Quality appraisal assessment for the included studies.

This indicates that the included studies met good reporting standards for health economic evaluations. However, several methodological concerns were identified, particularly regarding the estimation of effectiveness outcomes. Two studies relied on simulation models [37,38], and two estimated effectiveness using literature sources [35] or secondary data [40]. Adjustment of costs and outcomes for different timing (discounting) was unclear in two studies [35,38] and not applicable in three studies [39,41,42] due to the short analytic time horizon. While most included studies considered uncertainty, one study only partially addressed this by acknowledging a source of uncertainty arising from a simplifying assumption but did not further explore it in the evaluation [38]. These methodological limitations should be considered when interpreting the evidence from this review.

### 3.3. Geographical distribution of NCD economic evaluations

Of the seven studies, three were conducted in high-income countries and focused on natural disasters [35,37,38] while the remaining four were conducted in lower-middle-income countries (Lebanon, Gaza, Kosovo, and Pakistan) [39–42] and all addressed conflict-related crises. The high-income studies were undertaken in British Columbia province, Canada, addressing a wildfire (n = 1) [35], and in the United States (n = 2) [37,38]. The US-based studies included one in New York City that examined post-Hurricane Sandy natural disasters [37], and the second was conducted in rural North Carolina and addressed wildfire disasters [38].

The classification of countries’ income levels is based on the World Bank’s Gross National Income per capita classification for the year the article was published [43].

### 3.4. Economic evidence of NCD management in humanitarian contexts

Regarding the types of health economic evaluations, five studies exclusively applied cost-effectiveness analyses [35,37,39,40,42], one conducted a cost-benefit analysis [38], and the last reported multiple health economic approaches, including cost-effectiveness, cost-utility, and partial cost-benefit analyses [41]. However, upon review of the latter article, only the cost-effectiveness results were clearly reported [41]. Of the seven studies, four used a healthcare system perspective [35,37,40,42] and three adopted a societal perspective [38,39,41].

The included studies employed a range of methodological approaches, with a focus on model-based analyses that integrate data from multiple sources and enable comprehensive uncertainty assessment. One study used a Markov model [35], the most common traditional method, while three studies used simulation modelling analyses [37,38,40]: micro-simulation [40], agent-based simulation [37], and forecast-based simulation [38]. Advanced simulation modelling is a powerful and cost-effective way to study complex interventions and heterogeneous populations. Nonetheless, simulation models are increasingly in demand in health decision modelling [44]. Three studies reported economic evaluations conducted alongside randomised controlled trials [39,41,42].

### 3.5. NCD interventions evaluated in humanitarian contexts

Among the NCDs addressed in the existing health economic evaluations, mental health disorders, mainly depression, anxiety and stress-related disorders, were the most frequently reported, appearing in four studies [37,39,41,42]. Two studies addressed asthma [35,38], one of which also evaluated congestive heart failure [38]. One study examined a range of public health interventions [40], including tobacco bans, asthma treatment, breast cancer mammography screening and early-stage management, and early-stage colorectal cancer treatment, and incorporated diabetes as an NCD burden model without cost-effectiveness analysis.

The type of intervention varied across records, depending on the context and type of humanitarian crisis, as listed in Table 2. Four of the seven studies were treatment-focused on mental health disorders, while two were prevention-focused on asthma disorders due to wildfires. One study had a mixed focus on the efficiency of multiple public interventions for the prevention and treatment of predefined NCDs.

Overall, the review results demonstrate limited available evidence on the economic evaluation of NCD interventions in humanitarian contexts. Although the small number of included studies precludes broad generalisations about the wider evidence base, the studies identified in this review suggest an uneven geographical distribution, variation in crisis typology and variation in methodological approaches. Most included studies originated from conflict-affected contexts, focused on mental health conditions and utilised a cost-effectiveness economic approach as the predominant evaluation method. Notably, no included studies originated from low-income contexts, and evidence gaps remain for some of the highest-burden NCDs. Evidence relating to cancer was limited and confined to one study evaluating cancer screening and treatment as part of a broader package of public health interventions. No included study conducted a full health economic evaluation of diabetes specific interventions.

## 4. Discussion

### 4.1. Main findings, meaning and implications

This scoping review demonstrates that published full economic evaluations of interventions targeting NCDs in humanitarian contexts are scarce. The review identified a limited evidence base despite the growing burden of NCDs among crisis-affected populations and the substantial resource constraints facing humanitarian health systems. This finding highlights a marked mismatch between the scale of NCD-related needs in humanitarian contexts and the availability of robust economic evidence to inform resource allocation, programme design and policy decisions. The limited number of studies identified in this review is consistent with previous findings suggesting that health economic evidence in humanitarian contexts remains scarce. For example, Doocy et al. noted that health economic analyses account for only a small proportion of effectiveness studies evaluating humanitarian health interventions in LMICs, with minimal attention to NCDs [45]. This suggests that economic evidence may remain underutilised to inform prioritisation decisions, despite the high cost of humanitarian interventions, resource constraints, and the increasing need for health resources and commodities.

Given the small number of included studies, broad conclusions about the wider evidence base cannot be drawn. However, within the studies identified by this review, there was variation in geographical setting, crisis type, disease focus and methodological approach. No eligible studies were identified from low- income countries, even though these settings bear the highest burden of humanitarian crises and a disproportionate NCD burden [46]. This highlights an evidence gap in terms of the availability and use of published economic evidence for NCDs in humanitarian contexts in low-income countries. This is notable as low-income settings can have more resource-constrained and disrupted health systems than those represented in the evidence base identified in this study, including greater operational challenges, reduced health system capacity, and the affected population may also differ substantially.

The small number of eligible published studies indicates that decision-makers have limited published economic evidence specific to NCD interventions in humanitarian contexts. This does not imply that decision-making is based solely on published economic evaluations; routine programme data, unpublished analyses, and expert judgement may also inform resource-allocation decisions in these settings.

The conceptual framework used in this review links humanitarian crises and health-system disruption to NCD service delivery, economic evaluation and resource allocation decisions (Fig 1). Viewed through this framework, the limited evidence identified by the review occurs across several parts of this pathway, from NCD management in disrupted health systems to the generation of economic evidence for decision-making. This suggests that limitations in the current evidence base may affect not only assessments of the intervention’s value but also understanding of how contextual factors may influence the implementation of effective NCD programmes in humanitarian contexts.

The observed evidence gap may also partly reflect the operational and methodological challenges of conducting economic evaluations of NCD interventions in humanitarian contexts, including limited data availability, funding priorities, and security constraints that may limit the feasibility of such studies [47]. This emphasises the importance of developing context-specific economic evaluations that capture the operational realities of humanitarian and conflict-affected settings, and of greater investment in local research capacity to facilitate such evaluations. Where feasible, economic evidence on the costs and consequences of maintaining essential NCD services may help inform preparedness, service planning and resource-allocation decisions in humanitarian settings. The included studies reported that a range of research, governmental, international development, philanthropic, and public-sector funding sources supported the economic evaluations. These funding data describe the sources reported by the included publications but do not establish who commissioned or implemented the evaluations, who funds the underlying NCD programmes, or how these roles interacted within the humanitarian response. However, this review did not systematically examine who commissioned or implemented the evaluations, or how these roles related to funding and delivering the underlying NCD programmes. Further research is therefore needed to understand how economic evaluation can be feasibly commissioned, funded and embedded alongside NCD programmes in humanitarian contexts.

The variations in the types of humanitarian crises represented in the included studies may raise questions about whether different crisis typologies influence the applicability and methodological requirements of health economic evaluation of NCD interventions. Because natural disasters and conflicts differ fundamentally in duration, infrastructure damage, population displacement, and the level of disruption to the health system [48], the contextual factors influencing the conduct and interpretation of health economic evaluation may also differ. However, the small number of included studies limits conclusions about how crisis type may affect methodological approaches and economic outcomes. As a result, further research is required to explore whether different humanitarian crisis typologies, require distinct methodological approaches to evaluate NCD interventions.

Previous reviews in humanitarian contexts have reported that the health economics evidence base is often dominated by economic burden, cost analyses, and health financing studies, with comparatively few studies providing full health economic evaluations to inform resource allocation decisions [18,45]. Although all included studies conducted full economic evaluations (as per the inclusion criteria), the depth and methodological rigour of these evaluations varied considerably. In particular, relatively few studies employed cost–utility or cost–benefit analyses that explicitly link costs to health outcomes. This finding aligns with previous literature highlighting the scarcity of robust full economic evaluations that explicitly assess intervention value beyond cost estimates alone [16]. As a result, this evidence gap may provide only a partial basis for comparing alternative NCD interventions and may constrain decision-makers’ ability to prioritise interventions efficiently, ultimately increasing the risk of suboptimal allocation of scarce resources. Expanding the use of cost–utility and cost–benefit analyses in NCD policy and programme planning may support more comprehensive assessments of intervention value in resource-constrained settings [49].

Methodologically, model-based analyses are likely to be a pragmatic choice and may be particularly valuable in humanitarian contexts, given the difficulty of conducting primary research and obtaining reliable data because of population displacement, insecurity, and disrupted health information systems [18]. However, despite these advantages, applying complex health economic methods in low-resource settings can be extremely challenging because of limited data infrastructure, uncertainty in model inputs, and the frequent need to draw on evidence from other settings. As a result, model parameters may not fully represent the real context, which may influence the validity and transferability of findings [50,51]. Nevertheless, using available data may still be justified if its limitations are recognised and explored through sensitivity and uncertainty analyses. Where primary data are scarce, a model-based approach can inform decision-making, provided that uncertainty surrounding model inputs and assumptions is explicitly reported [52], as in some cases the choice not to use available data may result in no evidence being generated, even of low quality. Therefore, improvements are needed to strengthen model inputs using context-specific data, to improve transparency about modelling assumptions and uncertainty where possible, and to explore hybrid approaches that combine limited primary data collection with modelling. In addition, expanding the use of models that capture the complexity of humanitarian contexts, while ensuring that evidence quality is not compromised when primary data are limited will help generate robust economic evidence.

The included studies comprised analyses from both healthcare-system and societal perspectives. However, the cost items included in societal perspective analyses differed across studies (see data extraction table), and as such the inclusion of relevant patient costs was limited in some cases. In health economics, the term “perspective” refers to the viewpoints adopted by researchers to define the types of costs and outcomes considered in their records. Common perspectives include payer, healthcare system, and societal perspectives, each capturing distinct components and implications [53]. Healthcare perspective analyses primarily include direct health care costs related to intervention delivery and healthcare use, e.g., staff salaries, healthcare visits, equipment, and non-personnel costs such as programme administration [54]. By contrast, societal perspectives incorporate both direct and indirect costs, such as productivity losses, absenteeism, presenteeism, and changes in income [54].

As a result, the study perspective is important in shaping the assumptions of economic evaluations. It can also affect subsequent recommendations and policy decisions when compared with cost-effectiveness benchmarks/thresholds. No universally accepted perspective for economic evaluations exists [53], and the choice of perspective depends on the context, the evaluation question, the type of analysis being conducted, and the needs of the decision-maker [53,55]. However, in such humanitarian contexts, instability, prolonged displacement, and a disrupted health system exacerbate indirect costs, while many NCD cases entail long-term productivity losses. Therefore, limited consideration of broader societal and indirect costs may understate the full economic burden of NCDs on individuals and households, including the risk of catastrophic health expenditure.

This limitation is particularly important in settings where access to affordable medical care is limited, and financial protection mechanisms are weak. In humanitarian contexts, people rely heavily on out-of-pocket payments to meet their essential health needs. This reliance on out-of-pocket spending often leads to financial hardship and impoverishment, particularly in low- and middle-income countries [56,57]. Failure to incorporate these costs into economic evaluations obscures the full extent of the burden on households and reduces the relevance of findings for policy decision-making. Future research should therefore prioritise broader analytical perspectives that capture a wider range of societal costs, including productivity losses and quality-of-life impacts, where feasible.

Mental health interventions were more frequently represented in the studies identified in this review than interventions addressing other NCDs. This was particularly notable for conditions that contribute substantially to global morbidity and mortality, among crisis-affected populations such as diabetes, hypertension, cancers, chronic obstructive pulmonary disease and multimorbidity [58]. This imbalance is consistent with previous literature indicating that NCDs may remain overlooked and underrepresented in humanitarian contexts [59,60], potentially limiting the extent to which available evidence captures the broader burden of NCDs in these contexts. This imbalance creates a potential evidence gap that decision-makers need to guide resource allocation, budget planning, decisions on essential medicines, and long-term NCD programming in protracted crises. The emphasis on mental health may partly reflect broader humanitarian priorities, where psychosocial support has traditionally been more established than chronic NCD management. However, the limited published evidence on economic evaluations of other NCD conditions highlights a need for further economic research to better reflect the burden of NCDs in humanitarian contexts. This is particularly important in such contexts where disrupted access to healthcare may increase NCD risk and amplify the interconnection between chronic mental and physical health conditions [61]. Generating economic evidence that addresses a broader range of NCD conditions across humanitarian contexts may support future decision-making on investment priorities in humanitarian health programmes.

Overall, the findings of this review indicate that the available evidence on the economic evaluation of NCD interventions in humanitarian contexts is insufficient to support robust long-term planning and resource allocation.

In some included studies, relatively short follow-up periods limited the assessment of longer-term costs and outcomes. Where appropriate and feasible, future economic evaluations could consider longer analytic horizons or modelling approaches to explore longer-term consequences, while making assumptions and uncertainty explicit. This is particularly important for NCD interventions, given the long-term nature of NCDs and the protracted nature of humanitarian crises. As the health and economic benefits of NCD interventions accrue over time, short-term evaluations may underestimate the true costs and benefits. Further research should prioritise long-term economic modelling approaches to better capture sustained health outcomes and long-term resource implications in humanitarian contexts.

### 4.2. Limitations and gaps

#### 4.2.1. Limitations of included studies.

The limitations and gaps in applying health economic evaluations of NCD interventions in humanitarian contexts range from short time horizons to scalability issues, as summarised in Fig 3.

**Fig 3:**
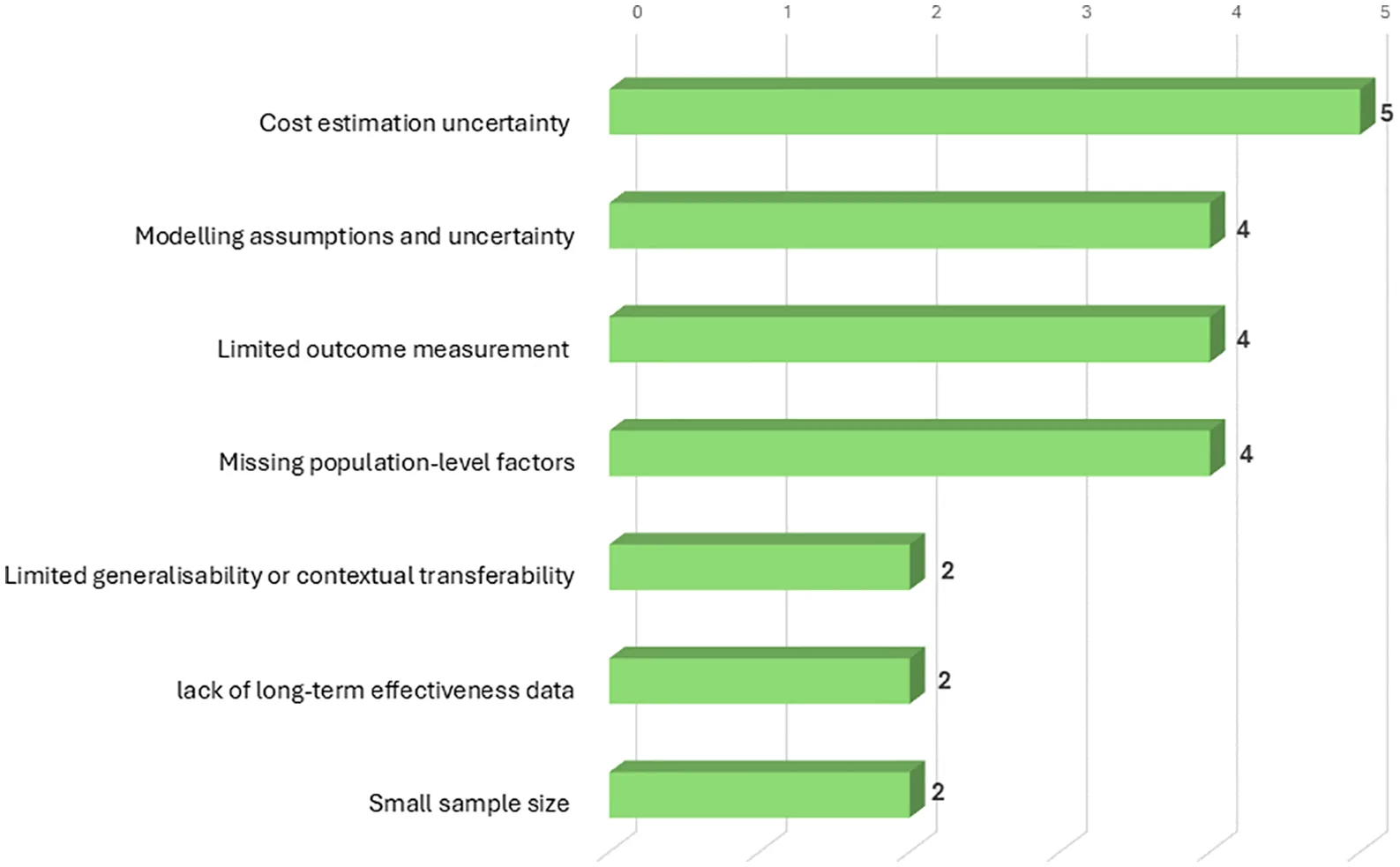
Limitations of the eligible studies.

Across the seven included records, the most frequently reported limitations were uncertainty in cost estimation, including inflation or omitted costs (n = 5) [37–40,42], followed by modelling assumptions and uncertainty (n = 4) [35,37,38,40] and incomplete outcome measurement, including the exclusion of broader societal benefits or quality-adjusted life years (n = 4). In addition, four studies highlighted the omission of important population-level factors such as demographic, geographic, or social determinants of health [35,37,38,40]. A smaller number of studies identified concerns related to limited generalisability or contextual transferability of findings (n = 2) [35,40] and short follow-up periods that restricted assessment of long-term effectiveness (n = 2) [39,41]. Two studies noted issues relating to attrition or small sample size (n = 2) [39,41].

Although the methodological quality assessment found that all included studies met most of the Drummond 10-item checklist, some limitations were identified that may reduce transparency and the transferability of findings across contexts. These limitations should be taken into account in the interpretation of the evidence.

#### 4.2.2. Limitations of the review process.

This review used a broad search strategy across seven major database and grey- literature sources. However, several limitations may have affected the completeness of the evidence identified. Publication and retrieval bias cannot be ruled out, as relevant evaluations may not have been published, indexed in the databases searched, identified by the search strategy, or publicly accessible. The findings should therefore be interpreted as representing the evidence identified through the review methods rather than the complete body of economic evidence that may exist in humanitarian settings.

First, although no language restrictions were applied in order to be as inclusive as possible, which is a strength of the study, all studies included in the final screening were published in English. Although some records were identified in non-English languages, none met the eligibility criteria during the title and abstract screening phase. While this reflects the current evidence, the potential for language bias cannot be entirely ruled out, as some relevant reports published in other languages may have been missed or uncaptured.

Second, the review searched seven major databases and grey literature covering health, humanitarian contexts, and health economic evaluation, thereby maximising the likelihood of capturing relevant records. Nevertheless, the possibility of publication and retrieval bias can’t be entirely excluded. Some relevant studies or reports may not be indexed, may have been difficult to identify through the available search mechanisms, or may not have been publicly accessible. Therefore, the findings should be interpreted as reflecting the body of evidence retrievable through the review methods employed. In addition, some sources, such as Google Scholar and the NHS Economic Evaluation Database, were not searched. The NHS Economic Evaluation Database has not been updated since 2015, which means it doesn’t cover contemporary economic evaluation evidence [62].

Third, the scope was intentionally restricted to conflict-related and natural crisis humanitarian contexts. As a result, studies conducted in pandemic and technological disaster settings were excluded. Excluding pandemics allowed for a more focused scope; however, it may limit the applicability of the results to pandemic-related disruptions in NCD care. Similarly, although technological disasters may meet the broader definition of a humanitarian context, they were not systematically incorporated into the search strategy and were excluded to maintain methodological consistency in the review scope and eligibility criteria. Further research is still needed to address the application of health economic evaluation to assess NCD management in pandemic and technological-disaster contexts.

Fourth, the review included only full health economic evaluations of NCD interventions in humanitarian contexts, examining records that capture both costs and outcomes. Consequently, partial health economic evaluations, such as cost analyses, as well as literature on health system financing and strengthening and on universal health coverage, were outside the review’s predefined scope. This may have led to the omission of relevant evidence on costs and resource use. However, this decision ensured conceptual clarity and consistency with the review objectives. Therefore, the findings should be interpreted as reflecting the available evidence on full health economic evaluations of NCD interventions rather than the broader economic literature relevant to humanitarian contexts.

Fifth, studies focusing on refugees and asylum seekers in stable, non-humanitarian contexts were excluded. Although these populations may face significant barriers to accessing NCD care and represent an important area for health economic evaluation, their context may differ substantially from that in acute humanitarian crises. This review therefore focused on interventions delivered during active emergencies, where health system capacity, resource availability, and policy environments differ markedly from those in more stable settings. Future research should consider dedicated analyses of NCD interventions for displaced populations living in non-emergency contexts to complement this evidence base.

Finally, this review focused on health economics studies evaluating NCD programmes and interventions in humanitarian contexts. It therefore did not capture economic evaluations of broader health programmes and other health priorities, such as communicable diseases. As a result, the review may not fully represent the overall health economic evidence generated in humanitarian contexts. However, this focused approach provides a more precise, in-depth assessment of health economic evidence on NCDs in these contexts.

#### 4.2.3. Evidence gaps identified through synthesis.

The synthesis was constrained by the limited available evidence. As only seven articles were included, this limited opportunities for comparison across different contexts, reduced the ability to identify consistent patterns within the evidence base, and restricted the depth of interpretation that could be obtained from the evidence.

## 5. Conclusion

This review highlighted a small number of published full health economic evaluations of NCD interventions in humanitarian contexts. The evidence identified was fragmented across geographical settings, NCD conditions and methodological approaches, limiting the extent to which firm conclusions can be drawn regarding policy decisions and optimal resource allocation in humanitarian NCD care.

Despite the limited number of identified studies, this review provides a valuable synthesis of existing full economic evaluations of NCD interventions, highlights underexplored areas, and helps identify priorities for future research.

The findings highlight the potential value of strengthening the availability and use of contextually relevant economic evidence for NCD decision-making during humanitarian emergencies. They lay the foundation for future research using contextually appropriate and methodologically robust approaches, including broader cost perspectives. Several evidence gaps were identified in the included records, including the absence of eligible studies from low-income countries and limited economic evidence for major NCDs such as cancer or diabetes interventions.

Some included studies were limited by short time horizons, incomplete consideration of broader societal costs, and reliance on data from other settings. Addressing these limitations may strengthen the relevance of future economic evidence on NCD interventions in humanitarian contexts.

The review also highlights the need for further research across diverse humanitarian contexts to deepen understanding of how contextual variation shapes the health economic evaluation of NCD programmes and interventions. Priority should be given to evaluating interventions targeting high-burden NCDs, developing contextually appropriate health economic methods, and generating evidence from underrepresented settings, particularly low-income and highly disrupted humanitarian contexts. Strengthening the evidence base in these areas may support more informed, effective, equitable and sustainable policies and funding strategies that better integrate NCD care into humanitarian health responses.

## Supporting information

S1 Checklist PRISMA_ScR checklist. This supplementary table presents the completed PRISMA extension for the scoping review checklist.
(DOCX)

S1 Text Scoping review search strategy. This supplementary file illustrates the three concepts: humanitarian contexts, noncommunicable diseases and economic evaluation, including their MeSH terms and additional keywords used in the seven databases search.
(DOCX)

S1 Table Grey literature search strategy. This supplementary table presents the two main websites used for grey literature searches, clarifying the search terms applied and the terms selected as filters via the available drop-down menu on each website.
(DOCX)

S2 Table Data charting and extraction framework. This supplementary table demonstrates the complete datasets extracted from each study. Data were systematically charted across predefined domains, including study identification, study characteristics, intervention and comparator details, outcomes measured, economic evaluation characteristics, key findings, and quality assessment information.
(XLSX)

S3 Table Quality appraisal assessment for the included studies. This table illustrates the quality assessment of included economic evaluations using the Drummond 10-item checklist.
(XLSX)
