# Supplementary material for: Economic evaluations of interventions for the treatment and management of non-communicable diseases in humanitarian contexts: A scoping review
Source: PLOS Glob Public Health. 2026 Nov 11;6(11):e0006740. doi: 10.1371/journal.pgph.0006740 (PMC13754108; doi:10.1371/journal.pgph.0006740)
Supplement: Supplementary file 2 [file pgph.0006740.s002.docx]

**S1 Text. Scoping review search strategy**

## CAB Abstracts <1910 to 2026 Week 27>

1	exp Health care cost/ or exp cost benefit analysis/ or exp economic evaluation/ or exp economic analysis/	132319

2	(Health adj2 (cost* or analys* or outcome* or expen* or financ*or model*)).tw.	59012

3	(Economic adj2 (evaluat* or analys*)).tw.	72500

4	(cost adj2 (evaluat* or analys* or effectiv* or utilit* or benefit* or minimiz*)).tw.	155206

5	(value adj1 (money or monetary)).tw.	2028

6	(incremental adj3 ratio*).tw.	2615

7	(ICER or "quality adjusted life year*" or QALY*).tw.	3421

8	(resource adj3 (allocat* or priorit*)).tw.	17252

9	(decision adj2 (tree or tool* or analys*)).tw.	20726

10	or/1-9	323570

11	exp hypertension/ or exp noncommunicable diseases/ or exp diabetes mellitus/ or exp cardiovascular diseases/ or exp chronic diseases/	271865

12	exp respiratory diseases/ or exp asthma/ or exp chronic obstructive pulmonary disease/	312993

13	exp depression/ or exp mental disorders/ or exp anxiety/ or exp psychoses/	103481

14	nervous system disease/	33783

15	exp Cancer/	1983

16	exp Cancer/	1983

17	((noncommunicable or non-communicable or noninfectious or Non-infectious or Lifestyle or Chronic) adj2 (disease* or condition* or disorder*)).tw.	100564

18	((cardiovascular or heart or lung or pulmunary) adj2 (disease* or condition* or disorder*)).tw.	319740

19	((peripheral arterial or aortic or cerebrovascular) adj2 (disease* or condition* or disorder*)).tw.	9340

20	(stroke or hypertension or angina or heart attack* or myocardial infarction or heart failure).tw.	107456

21	NCD*.tw.	7334

22	(Cancer* or Tumo?r* or malignan* or carcinoma or oncology or metastasis or neoplas*).tw.	475063

23	(asthma or respiratory or pulmunary or COPD).tw.	396813

24	((mental* or psych* or anxiety or bipolar or borderline personality or obsessive compulsive) adj2 (disorder* or disease* or condition*)).tw.	70834

25	(Alzheimer or dementia* or Parkinson* or multiple sclerosis or migraine or headache*or motor neuron).tw.	27780

26	or/11-25	1277118

27	exp crisis/ or exp emergency relief/ or exp disasters/ or exp natural disaster/	67491

28	(emergency adj3 (setting* or context* or countr* or work or relief or program* or area*)).tw.	4010

29	(fragile adj3 (setting* or area* or context* or state*)).tw.	1752

30	(crisis adj3 (setting or state)).tw.	394

31	(conflict adj3 (area* or zone* or setting*)).tw.	2591

32	(mass adj3 (incident* or violence or execution* or casualt*)).tw.	488

33	(disaster* or catastrophe* or cataclysmic).tw.	55964

34	(humanitarian or refugee* or torture).tw.	14967

35	(Natural adj3 (crisis or devastation or disaster*)).tw.	35713

36	(drought* or earthquake* or evacuation* or famine* or flood* or hurricane or cyclone* or landslide* or "land slide*" or tsunami* or "tidal wave*" or volcano* or wildfire* or "wild fire*").tw.	416128

37	(Man-made adj3 (crisis or crises)).tw.	23

38	(armed adj2 (conflict* or war*)).tw.	1791

39	genocide.tw.	444

40	or/27-39	459119

41	10 and 26 and 40	878

## ECONLIT

S3	S1 AND S2	Search modes - Proximity	Interface - EBSCOhost Research Databases

Search Screen - Advanced Search

Database - EconLit	87

S2	TI ( "noncommunicable disease*" OR "non-communicable disease*" OR "noninfectious disease*" OR "non-infectious disease*" OR "lifestyle disease*" OR "chronic disease*" OR NCD* OR "cardiovascular disease*" OR "heart disease*" OR "lung disease*" OR "pulmonary disease*" OR "peripheral arterial disease*" OR "aortic disease*" OR "cerebrovascular disease*" OR stroke OR hypertension OR angina OR "heart attack*" OR "myocardial infarction" OR "heart failure" OR cancer* OR tumo?r* OR malignan* OR carcinoma OR oncology OR metastasis OR neoplas* OR asthma OR diabetes OR COPD OR "mental disorder*" OR "psych* disorder*" OR "anxiety disorder*" OR "bipolar disorder*" OR "borderline personality disorder*" OR "obsessive compulsive disorder*" OR Alzheimer OR dementia* OR Parkinson* OR "multiple sclerosis" OR migraine OR headache* OR "motor neuron" )) ) OR AB ( "noncommunicable disease*" OR "non-communicable disease*" OR "noninfectious disease*" OR "non-infectious disease*" OR "lifestyle disease*" OR "chronic disease*" OR NCD* OR "cardiovascular disease*" OR "heart disease*" OR "lung disease*" OR "pulmonary disease*" OR "peripheral arterial disease*" OR "aortic disease*" OR "cerebrovascular disease*" OR stroke OR hypertension OR angina OR "heart attack*" OR "myocardial infarction" OR "heart failure" OR cancer* OR tumo?r* OR malignan* OR carcinoma OR oncology OR metastasis OR neoplas* OR asthma OR diabetes OR COPD OR "mental disorder*" OR "psych* disorder*" OR "anxiety disorder*" OR "bipolar disorder*" OR "borderline personality disorder*" OR "obsessive compulsive disorder*" OR Alzheimer OR dementia* OR Parkinson* OR "multiple sclerosis" OR migraine OR headache* OR "motor neuron" )) )	Search modes - Proximity	Interface - EBSCOhost Research Databases

Search Screen - Advanced Search

Database - EconLit	6,447

S1	TI ( ( disaster* OR catastrophe* OR refugee* OR humanitarian OR "emergency setting*" OR "emergency context*" OR "emergency countr*" OR "emergency work" OR "emergency relief" OR "emergency program*" OR "emergency area*" OR "fragile setting*" OR "fragile area*" OR "fragile context*" OR "fragile state*" OR "crisis setting" OR "crisis state" OR "conflict area*" OR "conflict zone*" OR "conflict setting*" OR "mass incident*" OR "mass violence" OR "mass execution*" OR "mass casualt*" OR "natural crisis" OR "natural devastation" OR "natural disaster*" OR drought* OR earthquake* OR evacuation* OR famine* OR flood* OR hurricane OR cyclone* OR landslide* OR "land slide*" OR tsunami* OR "tidal wave*" OR volcano* OR wildfire* OR "wild fire*" OR "man-made crisis" OR "man-made crises" OR "armed conflict*" OR "armed war*" OR genocide )) ) OR AB ( ( disaster* OR catastrophe* OR refugee* OR humanitarian OR "emergency setting*" OR "emergency context*" OR "emergency countr*" OR "emergency work" OR "emergency relief" OR "emergency program*" OR "emergency area*" OR "fragile setting*" OR "fragile area*" OR "fragile context*" OR "fragile state*" OR "crisis setting" OR "crisis state" OR "conflict area*" OR "conflict zone*" OR "conflict setting*" OR "mass incident*" OR "mass violence" OR "mass execution*" OR "mass casualt*" OR "natural crisis" OR "natural devastation" OR "natural disaster*" OR drought* OR earthquake* OR evacuation* OR famine* OR flood* OR hurricane OR cyclone* OR landslide* OR "land slide*" OR tsunami* OR "tidal wave*" OR volcano* OR wildfire* OR "wild fire*" OR "man-made crisis" OR "man-made crises" OR "armed conflict*" OR "armed war*" OR genocide )) )	Search modes - Proximity	Interface - EBSCOhost Research Databases

Search Screen - Advanced Search

Database - EconLit	20,162

## Embase <1996 to 2026 Week 26>

1	exp "costs and cost analysis"/ or exp economics, medical/	1123218

2	exp Models, Economic/	5423

3	quality-adjusted life years/	43947

4	(Health adj2 (cost* or analys* or outcome* or expen* or financ*or model*)).tw.	230185

5	(Economic adj2 (evaluat* or analys*)).tw.	40898

6	(cost adj2 (evaluat* or analys* or effectiv* or utilit* or benefit* or minimiz*)).tw.	341219

7	(value adj1 (money or monetary)).tw.	1301

8	(incremental adj3 ratio*).tw.	24834

9	(ICER or "quality adjusted life year*" or QALY*).tw.	44379

10	(resource adj3 (allocat* or priorit*)).tw.	25698

11	(decision adj2 (tree or tool* or analys*)).tw.	67602

12	or/1-11	1525113

13	exp Diabetes Mellitus/ or Noncommunicable Diseases/ or exp Chronic Disease/	1648270

14	exp Cardiovascular Diseases/	5294253

15	exp asthma/ or exp pulmonary disease, chronic obstructive/	469966

16	exp Mental Disorders/ or Depression/	2910347

17	exp Nervous System Diseases/	4653553

18	exp neoplasms/	5819334

19	((noncommunicable or non-communicable or noninfectious or Non-infectious or Lifestyle or Chronic) adj2 (disease* or condition* or disorder*)).tw.	597977

20	((cardiovascular or heart or lung or pulmunary) adj2 (disease* or condition* or disorder*)).tw.	778613

21	((peripheral arterial or aortic or cerebrovascular) adj2 (disease* or condition* or disorder*)).tw.	80060

22	(stroke or hypertension or angina or heart attack* or myocardial infarction or heart failure).tw.	1751458

23	NCD*.tw.	21344

24	(Cancer* or Tumo?r* or malignan* or carcinoma or oncology or metastasis or neoplas*).tw.	5659498

25	(asthma or diabetes or COPD).tw.	1473748

26	((mental* or psych* or anxiety or bipolar or borderline personality or obsessive compulsive) adj2 (disorder* or disease* or condition*)).tw.	380673

27	(Alzheimer or dementia* or Parkinson* or multiple sclerosis or migraine or headache*or motor neuron).tw.	672119

28	or/13-26	16673835

29	exp disasters/ or refugees/	53560

30	(emergency adj3 (setting* or context* or countr* or work or relief or program* or area*)).tw.	24866

31	(fragile adj3 (setting* or area* or context* or state*)).tw.	1063

32	(crisis adj3 (setting or state)).tw.	721

33	(conflict adj3 (area* or zone* or setting*)).tw.	3256

34	(mass adj3 (incident* or violence or execution* or casualt*)).tw.	6821

35	(disaster* or catastrophe* or cataclysmic).tw.	49284

36	(humanitarian or refugee* or torture).tw.	27327

37	(Natural adj3 (crisis or devastation or disaster*)).tw.	8141

38	(drought* or earthquake* or evacuation* or famine* or flood* or hurricane or cyclone* or landslide* or "land slide*" or tsunami* or "tidal wave*" or volcano* or wildfire* or "wild fire*").tw.	123409

39	(Man-made adj3 (crisis or crises)).tw.	16

40	(armed adj2 (conflict* or war*)).tw.	2786

41	genocide.tw.	871

42	or/29-41	233204

43	12 and 28 and 42	5180

## Global Health <1973 to 2026 Week 27>

1	exp Health care cost/ or exp cost benefit analysis/ or exp economic evaluation/ or exp economic analysis/	52677

2	(Health adj2 (cost* or analys* or outcome* or expen* or financ*or model*)).tw.	92332

3	(Economic adj2 (evaluat* or analys*)).tw.	10639

4	(cost adj2 (evaluat* or analys* or effectiv* or utilit* or benefit* or minimiz*)).tw.	65895

5	(value adj1 (money or monetary)).tw.	428

6	(incremental adj3 ratio*).tw.	4056

7	(ICER or "quality adjusted life year*" or QALY*).tw.	6413

8	(resource adj3 (allocat* or priorit*)).tw.	7701

9	(decision adj2 (tree or tool* or analys*)).tw.	8382

10	or/1-9	168597

11	exp hypertension/ or exp noncommunicable diseases/ or exp diabetes mellitus/ or exp cardiovascular diseases/ or exp chronic diseases/	372714

12	exp respiratory diseases/ or exp asthma/ or exp chronic obstructive pulmonary disease/	441740

13	exp depression/ or exp mental disorders/ or exp anxiety/ or exp psychoses/	170120

14	nervous system disease/	29910

15	exp Cancer/	1298

16	exp Cancer/	1298

17	((noncommunicable or non-communicable or noninfectious or Non-infectious or Lifestyle or Chronic) adj2 (disease* or condition* or disorder*)).tw.	133425

18	((cardiovascular or heart or lung or pulmunary) adj2 (disease* or condition* or disorder*)).tw.	417681

19	((peripheral arterial or aortic or cerebrovascular) adj2 (disease* or condition* or disorder*)).tw.	17463

20	(stroke or hypertension or angina or heart attack* or myocardial infarction or heart failure).tw.	155854

21	NCD*.tw.	7775

22	(Cancer* or Tumo?r* or malignan* or carcinoma or oncology or metastasis or neoplas*).tw.	541880

23	(asthma or respiratory or pulmunary or COPD).tw.	477802

24	((mental* or psych* or anxiety or bipolar or borderline personality or obsessive compulsive) adj2 (disorder* or disease* or condition*)).tw.	125171

25	(Alzheimer or dementia* or Parkinson* or multiple sclerosis or migraine or headache*or motor neuron).tw.	40576

26	or/11-25	1537115

27	exp crisis/ or exp emergency relief/ or exp disasters/ or exp natural disaster/	12972

28	(emergency adj3 (setting* or context* or countr* or work or relief or program* or area*)).tw.	4252

29	(fragile adj3 (setting* or area* or context* or state*)).tw.	454

30	(crisis adj3 (setting or state)).tw.	165

31	(conflict adj3 (area* or zone* or setting*)).tw.	1700

32	(mass adj3 (incident* or violence or execution* or casualt*)).tw.	884

33	(disaster* or catastrophe* or cataclysmic).tw.	18245

34	(humanitarian or refugee* or torture).tw.	14499

35	(Natural adj3 (crisis or devastation or disaster*)).tw.	8926

36	(drought* or earthquake* or evacuation* or famine* or flood* or hurricane or cyclone* or landslide* or "land slide*" or tsunami* or "tidal wave*" or volcano* or wildfire* or "wild fire*").tw.	31483

37	(Man-made adj3 (crisis or crises)).tw.	14

38	(armed adj2 (conflict* or war*)).tw.	1435

39	genocide.tw.	275

40	or/27-39	61353

41	10 and 26 and 40	1221

## Ovid MEDLINE(R) ALL <1946 to July 02, 2026>

1	exp "costs and cost analysis"/ or exp economics, medical/	301159

2	exp Models, Economic/	17343

3	quality-adjusted life years/	19558

4	(Health adj2 (cost* or analys* or outcome* or expen* or financ*or model*)).tw.	185504

5	(Economic adj2 (evaluat* or analys*)).tw.	31101

6	(cost adj2 (evaluat* or analys* or effectiv* or utilit* or benefit* or minimiz*)).tw.	271830

7	(value adj1 (money or monetary)).tw.	1071

8	(incremental adj3 ratio*).tw.	15567

9	(ICER or "quality adjusted life year*" or QALY*).tw.	26670

10	(resource adj3 (allocat* or priorit*)).tw.	22370

11	(decision adj2 (tree or tool* or analys*)).tw.	54930

12	or/1-11	732408

13	exp Diabetes Mellitus/ or Noncommunicable Diseases/ or exp Chronic Disease/	1238609

14	exp Cardiovascular Diseases/	2959348

15	exp asthma/ or exp pulmonary disease, chronic obstructive/	220456

16	exp Mental Disorders/ or Depression/	1732788

17	exp Nervous System Diseases/	3167739

18	exp neoplasms/	4271163

19	((noncommunicable or non-communicable or noninfectious or Non-infectious or Lifestyle or Chronic) adj2 (disease* or condition* or disorder*)).tw.	426346

20	((cardiovascular or heart or lung or pulmunary) adj2 (disease* or condition* or disorder*)).tw.	577833

21	((peripheral arterial or aortic or cerebrovascular) adj2 (disease* or condition* or disorder*)).tw.	59897

22	(stroke or hypertension or angina or heart attack* or myocardial infarction or heart failure).tw.	1260441

23	NCD*.tw.	14102

24	(Cancer* or Tumo?r* or malignan* or carcinoma or oncology or metastasis or neoplas*).tw.	4665225

25	(asthma or diabetes or COPD).tw.	1015144

26	((mental* or psych* or anxiety or bipolar or borderline personality or obsessive compulsive) adj2 (disorder* or disease* or condition*)).tw.	293787

27	(Alzheimer or dementia* or Parkinson* or multiple sclerosis or migraine or headache*or motor neuron).tw.	494496

28	or/13-26	13512324

29	exp disasters/ or refugees/	139459

30	(emergency adj3 (setting* or context* or countr* or work or relief or program* or area*)).tw.	18712

31	(fragile adj3 (setting* or area* or context* or state*)).tw.	1081

32	(crisis adj3 (setting or state)).tw.	598

33	(conflict adj3 (area* or zone* or setting*)).tw.	3297

34	(mass adj3 (incident* or violence or execution* or casualt*)).tw.	5695

35	(disaster* or catastrophe* or cataclysmic).tw.	49255

36	(humanitarian or refugee* or torture).tw.	27654

37	(Natural adj3 (crisis or devastation or disaster*)).tw.	7706

38	(drought* or earthquake* or evacuation* or famine* or flood* or hurricane or cyclone* or landslide* or "land slide*" or tsunami* or "tidal wave*" or volcano* or wildfire* or "wild fire*").tw.	128272

39	(Man-made adj3 (crisis or crises)).tw.	19

40	(armed adj2 (conflict* or war*)).tw.	2917

41	genocide.tw.	879

42	or/29-41	293507

43	12 and 28 and 42	2392

## SCOPUS

( TITLE-ABS-KEY ( "emergency setting*" OR "emergency context*" OR "emergency countr*" OR "emergency work" OR "emergency relief" OR "emergency program*" OR "emergency area*" OR "fragile setting*" OR "fragile area*" OR "fragile context*" OR "fragile state*" OR "crisis setting" OR "crisis state" OR "conflict area*" OR "conflict zone*" OR "conflict setting*" OR "mass incident*" OR "mass violence" OR "mass execution*" OR "mass casualt*" OR disaster* OR catastrophe* OR cataclysmic OR humanitarian OR refugee* OR torture OR "natural crisis" OR "natural devastation" OR "natural disaster*" OR drought* OR earthquake* OR evacuation* OR famine* OR flood* OR hurricane OR cyclone* OR landslide* OR "land slide*" OR tsunami* OR "tidal wave*" OR volcano* OR wildfire* OR ‘‘wild fire*’’ OR "Man-made crisis" OR crises OR "armed conflict*" OR "armed war*" OR genocide ) AND TITLE-ABS-KEY ( "Health cost* " OR "health analys* " OR "health outcome* " OR "health expen* " OR "health financ*" OR "health model " OR "Economic evaluat* " OR "Economic analys* " OR "cost evaluat* " OR "cost analys* " OR "cost effectiv* " OR "cost utilit*" OR "cost benefit* " OR "cost minimiz* " OR "value for money " OR "monetary value " OR "incremental cost " OR "incremental ratio " OR ICER OR "quality adjusted life year* " OR QALY* OR "resource allocat* " OR "resource priorit* " OR "decision tree " OR "decision tool* " OR "decision analys*" ) AND TITLE-ABS-KEY ( "noncommunicable disease*" OR "non-communicable disease*" OR NCD OR "noninfectious disease*" OR "non-infectious disease*" OR "lifestyle disease*" OR "chronic disease*" OR "chronic condition*" OR "cardiovascular disease*" OR "cardiovascular condition*" OR "heart disease*" OR "lung disease*" OR "pulmonary disease*" OR "peripheral arterial disease*" OR "aortic disease*" OR "cerebrovascular disease*" OR stroke OR hypertension OR angina OR "heart attack*" OR "myocardial infarction" OR "heart failure" OR cancer* OR tumor* OR tumour* OR malignan* OR carcinoma OR oncology OR metastasis OR neoplas* OR asthma OR diabetes OR COPD OR "mental disease*" OR "mental disorder*" OR "mental condition*" OR "psychiatric disorder*" OR "anxiety disorder*" OR bipolar OR "borderline personality" OR "obsessive compulsive disorder*" OR "obsessive compulsive disease*" OR Alzheimer OR dementia* OR Parkinson* OR "multiple sclerosis" OR migraine OR headache* OR "motor neuron" ) ) = 290

## # Database: Web of Science Core Collection

\# Entitlements:

- WOS.SCI: 1900 to 2026

- WOS.AHCI: 1975 to 2026

- WOS.ESCI: 2021 to 2026

- WOS.ISTP: 1990 to 2026

- WOS.SSCI: 1900 to 2026

- WOS.ISSHP: 1990 to 2026

\# Searches:

1: TS=((	"health cost*" OR "health analys*" OR "health outcome*" OR "health expen*" OR "health financ*" OR "health model*"	OR "economic evaluat*" OR "economic analys*"	OR "cost evaluat*" OR "cost analys*" OR "cost effectiv*" OR "cost utilit*" OR "cost benefit*" OR "cost minimiz*"	OR "value of money" OR "value monetary"	OR "incremental ratio*" OR ICER	OR "quality adjusted life year*" OR QALY*	OR "resource allocat*" OR "resource priorit*"	OR "decision tree" OR "decision tool*" OR "decision analys*" ))	Results: 940716

2: TS=((	"noncommunicable disease*" OR "non-communicable disease*" OR "noninfectious disease*" OR "non-infectious disease*" OR "lifestyle disease*" OR "chronic disease*" OR NCD*	OR "cardiovascular disease*" OR "heart disease*" OR "lung disease*" OR "pulmonary disease*"	OR "peripheral arterial disease*" OR "aortic disease*" OR "cerebrovascular disease*"	OR stroke OR hypertension OR angina OR "heart attack*" OR "myocardial infarction" OR "heart failure"	OR cancer* OR tumo?r* OR malignan* OR carcinoma OR oncology OR metastasis OR neoplas*	OR asthma OR diabetes OR COPD	OR "mental disorder*" OR "psych* disorder*" OR "anxiety disorder*" OR "bipolar disorder*" OR "borderline personality disorder*" OR "obsessive compulsive disorder*"	OR Alzheimer OR dementia* OR Parkinson* OR "multiple sclerosis" OR migraine OR headache* OR "motor neuron" ))	Results: 9337111

3: TS=((	disaster* OR catastrophe* OR refugee* OR humanitarian	OR "emergency setting*" OR "emergency context*" OR "emergency countr*" OR "emergency work" OR "emergency relief" OR "emergency program*" OR "emergency area*"	OR "fragile setting*" OR "fragile area*" OR "fragile context*" OR "fragile state*"	OR "crisis setting" OR "crisis state"	OR "conflict area*" OR "conflict zone*" OR "conflict setting*"	OR "mass incident*" OR "mass violence" OR "mass execution*" OR "mass casualt*"	OR "natural crisis" OR "natural devastation" OR "natural disaster*"	OR drought* OR earthquake* OR evacuation* OR famine* OR flood* OR hurricane OR cyclone* OR landslide* OR "land slide*" OR tsunami* OR "tidal wave*" OR volcano* OR wildfire* OR "wild fire*"	OR "man-made crisis" OR "man-made crises"	OR "armed conflict*" OR "armed war*"	OR genocide ))	Results: 1052518

4: #1 AND #2 AND #3	Results: 1012
