# Supplementary material for: Economic evaluations of interventions for the treatment and management of non-communicable diseases in humanitarian contexts: A scoping review
Source: PLOS Glob Public Health. 2026 Nov 11;6(11):e0006740. doi: 10.1371/journal.pgph.0006740 (PMC13754108; doi:10.1371/journal.pgph.0006740)
Supplement: Supplementary file 3 [file pgph.0006740.s003.docx]

**S1 Table. Grey literature search strategy (May 2026)**

| **Website** | **Search terms** | **Filter used** | **No. of Articles** | **No. of Included** | **Excluded** |
| --- | --- | --- | --- | --- | --- |
| Reliefweb#  https://reliefweb.int/ | hypertension OR "noncommunicable diseases" OR diabetes OR cancer OR tumor OR pulmonary OR cardiovascular OR "chronic diseases" | Theme:  Humanitarian Financing | 43 | 0 | Not matching eligible criteria= 39  Duplicated= 4 |
|  | "noncommunicable diseases" OR "non infectious" OR "chronic diseases" | Theme:  Humanitarian Financing | 13 | 0 | Not matching eligible criteria=12  Duplicated= 1 |
|  | "Cardiovascular Diseases" OR hypertension | Theme:  Humanitarian Financing | 4 | 0 | Not matching eligible criteria=3  Duplicated= 1 |
|  | "Mental Disorders" OR Depression OR "Nervous System Diseases" | Theme:  Humanitarian Financing | 39 | 0 | Not matching eligible criteria=36  Duplicated= 3 |
|  | Asthma OR "lung diseases" | Theme:  Humanitarian Financing | 1 | 0 | Not matching eligible criteria=1 |
|  | Cancer OR tumor OR oncology | Theme:  Humanitarian Financing | 23 | 0 | Not matching eligible criteria=19  Duplicated= 4 |
| WHO IRIS  https://iris.who.int/ | Economic evaluation /  Cost effective | Subject:  noncommunicable diseases  Emergencies | 18 | 0 | Not matching eligible criteria=18 |
|  | Economic evaluation | Subject:  noncommunicable diseases  Disaster planning | 10 | 0 | Not matching eligible criteria=1  Duplicated= 9 |
|  | Economic evaluation | Subject:  chronic diseases  Emergencies | 1 | 0 | Duplicated= 1 |
|  | Cost effective | Subject:  chronic diseases  Emergencies | 3 |  | Not matching eligible criteria=2  Duplicated= 1 |
|  | Cost effective | Subject:  noncommunicable diseases  Disaster planning | 12 | 0 | Not matching eligible criteria=6  Duplicated= 6 |
